# Enclosing a pen in a postal questionnaire follow-up to increase response rate: a study within a trial

**DOI:** 10.3310/nihropenres.13324.2

**Published:** 2023-04-20

**Authors:** Caroline Fairhurst, Gillian Parkinson, Catherine Hewitt, Camila Maturana, Laura Wiley, Fiona Rose, David Torgerson, Jessica Hugill-Jones, Alison Booth, Laura Bissell, Garry Tew

**Affiliations:** 1 York Trials Unit, Department of Health Sciences, University of York, York, YO10 5DD, UK; 2 British Wheel of Yoga Qualifications, Sleaford, NG34 7RU, UK; 3 York St John University, York, YO31 7EX, UK

**Keywords:** study within a trial, pen, postal questionnaire, retention, randomised controlled trial, older people, multimorbidity

## Abstract

**Background:**

Poor response rates to follow-up questionnaires can adversely affect the progress of a randomised controlled trial and the validity of its results. This embedded ‘study within a trial’ aimed to investigate the impact of including a pen with the postal 3-month questionnaire completed by the trial participants on the response rates to this questionnaire.

**Methods:**

This study was a two-armed randomised controlled trial nested in the Gentle Years Yoga (GYY) trial. Participants in the intervention group of the GYY trial were allocated 1:1 using simple randomisation to either receive a pen (intervention) or no pen with their 3-month questionnaire (control). The primary outcome was the proportion of participants sent a 3-month questionnaire who returned it. Secondary outcomes were time taken to return the questionnaire, proportion of participants sent a reminder to return the questionnaire, and completeness of the questionnaire. Binary outcomes were analysed using logistic regression, time to return by Cox Proportional Hazards regression and number of items completed by linear regression.

**Results:**

There were 111 participants randomised to the pen group and 118 to the no pen group who were sent a 3-month questionnaire. There was no evidence of a difference in return rates between the two groups (pen 107 (96.4%), no pen 117 (99.2%); OR 0.23, 95% CI 0.02 to 2.19, p=0.20). Furthermore, there was no evidence of a difference between the two groups in terms of time to return the questionnaire (HR 0.90, 95% CI 0.69 to 1.18, p=0.47), the proportion of participants sent a reminder (OR 0.85, 95% CI 0.48 to 1.53, p=0.60) nor the number of items completed (mean difference 0.51, 95% CI -0.04 to 1.06, p=0.07).

**Conclusion:**

The inclusion of a pen with the postal 3-month follow-up questionnaire did not have a statistically significant effect on response rate.

## Introduction

Randomised controlled trials (RCTs) are one of the key tools used to analyse the effectiveness of a new treatment. However, poor recruitment and retention rates pose a serious threat to RCTs as they can render the results of the trial inconclusive, prolong the duration of the trial and can even lead to the trial being closed down early ^1^. Participants not completing follow-up data collection, can be very problematic for RCTs as it reduces power and, if differential between the arms, can introduce attrition bias ^2^.

Various strategies have been deployed to help maximise retention in RCTs ^3^. One such strategy is to include a pen when posting a follow-up questionnaire. This strategy is hypothesised to help improve retention response rates as it gives participants the means to complete the questionnaire while also making participants feel more inclined to return the questionnaire due to encouragement of positive reciprocal behaviour provided by the pen ^4^. A study within a trial (SWAT) aiming to investigate the impact of posting a pen with the 3-month follow-up participant questionnaire was embedded in the Gentle Years Yoga (GYY) trial ^5^.

### Previous evidence

The TRIAL FORGE initiative has published an evidence pack on the use of sending a pen with a trial questionnaire and/or study materials on response rate ( https://www.trialforge.org/resource/evidence-pack-retention-adding-a-pen-ret3/). Based on five prior RCTs ^6– 10^, they concluded that sending a pen probably increases retention and response rate (random effects meta-analysis pooled effect: increase in response rates of 1.9%, 95% CI 0.0% to 3.7%). We shall update this meta-analysis with our results. 

## Methods

### Study design

This SWAT was a two-armed RCT embedded in the GYY trial that aims to investigate the impact of the offer of participation in a 12-week Yoga programme on the health-related quality of life of older adults with multimorbidity in England and Wales ^5^. This study is being conducted by the York Trials Unit (YTU), University of York (recruitment complete and trial in follow-up at the time of writing; ISRCTN13567538, registered 18/03/2019 https://www.isrctn.com/ISRCTN13567538). The SWAT was registered with the Northern Ireland Network for Trials Methodology Research SWAT repository on 01/04/2019 (SWAT92; https://www.qub.ac.uk/sites/TheNorthernIrelandNetworkforTrialsMethodologyResearch/SWATSWARInformation/Repositories/SWATStore/). The GYY trial, and its embedded sub-studies, was funded by the National Institute for Health and Care Research (NIHR) Health Technology Assessment (HTA) Programme (ref 17/94/36) and received approval from the North East–York Research Ethics Committee on 24/04/2019 (19/NE/0072), and the Health Research Authority. 

### Participants

This study included participants allocated to the intervention arm of the GYY trial. Participants in the usual care arm of GYY were included in a different retention SWAT, namely the offer of a one-off GYY class at the end of their 12-month participation in the trial. This SWAT will be reported separately. For logistical reasons, participants were randomised into the SWAT immediately after being randomised into the intervention arm of the main trial, but only those sent their 3-month questionnaire are actually included in this SWAT. Participants were not informed in advance that they could be randomised into a SWAT to receive a pen with their 3-month questionnaire. This means that specific consent for the SWAT was not obtained; this was approved by the Research Ethics Committee as it was considered low risk. Written informed consent for the GYY main trial was obtained from all participants who took part.

### Intervention

**Figure 1.**
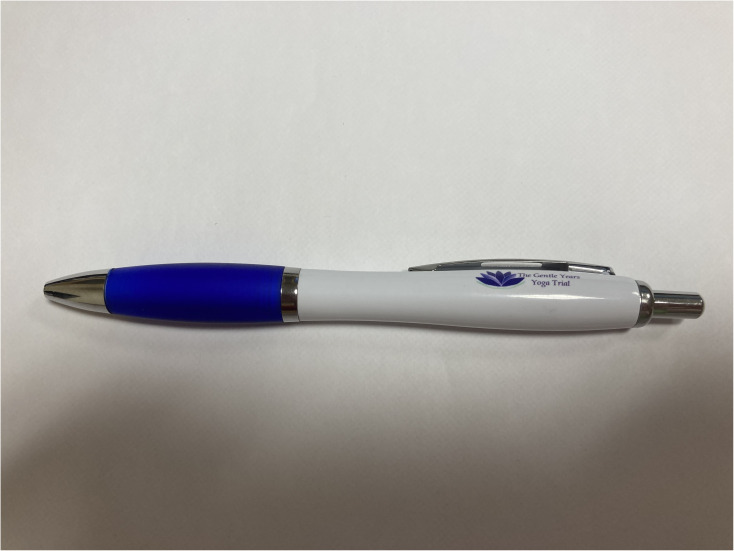
GYY logo-branded SWAT pen.

The 3-month questionnaire was a 16-page booklet containing the following questions and standardised instruments: EQ-5D-5L ^11^, PHQ-8 ^12^, GAD-7 ^13^, PROMIS-29 ^14^, UCLA 3-Item Loneliness scale ^15, 16^ and a direct loneliness question used in the English Longitudinal Study of Ageing, and questions asking about recent falls, health resource use, and participation in yoga over the previous 3 months. All participants in the GYY trial, who provided a valid mobile phone number and consented to be contacted *via* text message, were sent an SMS on the day the 3-month questionnaire was posted to them to pre-notify participants of its imminent arrival. Participants were also sent an unconditional GBP 5 with the questionnaire – this was in the form of cash (GBP 5 note) prior to the Covid-19 outbreak, and a shopping voucher thereafter. This action formed part of the host trial’s approach to promoting continuing participation and was standard practice with each follow-up questionnaire across both arms of the GYY trial. In addition, participants in the intervention group of the SWAT were sent a retractable ballpoint, black ink pen, branded with the GYY trial logo ( Figure 1) with their 3-month follow-up postal questionnaire whereas the control group were not sent a pen with their 3-month questionnaire. Participants who did not return their 3-month questionnaire within two weeks were sent a postal reminder questionnaire; pens were not sent with reminder notices in either group. Telephone reminders, up to a maximum of three phone calls per participant, were additionally employed if the 3-month questionnaire had still not been returned within two weeks of the reminder questionnaire being sent. 

### Sample size

No formal sample size calculation was undertaken as this was determined by the number of participants allocated to the intervention group of the main trial, which is typical for a SWAT. In this SWAT the 240 participants allocated to the intervention arm in the main trial were randomised; this sample size was sufficient to have 80% power to detect an increase in response rates from, for example, 80% in the ‘no pen’ group to 93% in the ‘pen’ group assuming 10% of participants withdraw before the 3-month follow-up timepoint.

### Randomisation

Participants were randomised using simple randomisation and a 1:1 allocation ratio. The trial statistician, not otherwise involved in the recruitment or follow-up of participants, generated the allocation sequence using Stata v15 (RRID: SCR_012763). Stata is a proprietary software but an open-access alternative in which the sequence could have been generated is Google Sheets (RRID:SCR_017679).

### Blinding

The nature of the intervention prevented the blinding of participants to their allocation. Nor were the statisticians analysing the data blinded, as the risk associated with this was deemed to be low as the outcomes are objective and were prespecified, along with the analyses, in the host trial protocol.

### Outcomes

The primary outcome of this SWAT was the proportion of sent out 3-month follow-up questionnaires that were returned. Secondary outcomes were time taken to return the questionnaire, the proportion of participants who were sent a reminder to complete the questionnaire, and the completeness of the questionnaire. A full list of the outcomes measured in this SWAT are detailed in Table 1.

**Table 1.** Outcome measures of the SWAT.

Outcome	Type	Definition
Proportion of 3-month questionnaires returned (primary)	Binary	The number of participants who returned their 3-month questionnaire divided by the number of participants who were sent this questionnaire.
Time taken to return 3-month questionnaire	Time to event	The number of days between the 3-month follow-up questionnaire being sent to the participant and being returned to York Trials Unit. This outcome is censored at 90 days for participants who do not return their 3-month questionnaire.
Reminder sent	Binary	The number of participants who were sent a reminder questionnaire to complete divided by the number of participants who were sent the 3-month questionnaire. Pens were not sent with the reminder questionnaires.
Number of items completed	Linear	The number of items completed in the questionnaire, if returned, out of a total of 78.

### Statistical analysis

Outcomes are summarised by group and overall. For binary outcome measures, the count and proportion are reported and mean and standard deviation for number of completed items. For time to return, the median survival time (from the Kaplan–Meier survivor function) and its 95% confidence interval (CI) are reported. Time to return was censored at 90 days (as participants were sent another follow-up questionnaire at 6 months post-randomisation) for participants who did not return their questionnaire.

Analyses were conducted under the principles of intention to treat (ITT) using two-tailed tests at the 5% significance level. Analyses were conducted in Stata v17 (RRID: SCR_012763). An open-access alternative that can perform an equivalent function to Stata for analysis is R, a free software environment for statistical computing and graphics (RRID: SCR_001905). The primary outcome of 3-month questionnaire response was analysed using logistic regression adjusting for SWAT group allocation (“pen” or “no pen”), age, gender and an indicator variable for if the participant was allocated to receive an intervention (pen and/or GBP 5 *versus* neither) in a previous 2×2 factorial SWAT, which was undertaken at the recruitment stage of the GYY trial ^17^. The treatment effect is estimated from the logistic regression model and presented as an odds ratio (OR) and adjusted absolute difference in proportions, with associated 95% CI and p-value. The secondary outcomes were analysed as follows: time to return 3-month questionnaire by Cox Proportional Hazards model, with treatment effect presented as a hazard ratio (HR); whether a reminder was sent by logistic regression, with treatment effect presented as an OR; and number of completed items by linear regression, with treatment effect presented as a mean difference. The models were adjusted as for the primary analysis.

*Post hoc* sensitivity analyses were conducted for the time to return the 3-month questionnaire outcome following indications that the Proportional Hazards assumption was violated for the Cox model. The analyses included both a log-rank test and a generalized gamma accelerated failure time (AFT) model, which are, respectively, a simpler and more complex alternative to the Cox model that do not assume proportional hazards. There was also evidence that the standardised residuals for the linear regression of number of items completed were not normally distributed and so the assumptions of this approach were questionable. Therefore, a post hoc Wilcoxon rank-sum test was performed, which does not make any assumption about the distribution.

27 participants in the pen group were not sent a pen with their questionnaire due to an administrative error; per-protocol (PP) analyses were additionally conducted by removing these participants from the analysis models.

## Results

In total, 240 participants were randomised into the intervention arm of the main GYY trial, and 229 (95.4%) participants were sent their 3-month questionnaire and so were included in this SWAT (pen n=111; no pen n=118). The remaining 11 participants withdrew from the main trial before 3 months and so were not sent any follow-up questionnaires (6 (5.1%) from the pen group, and 5 (4.1%) from the no pen group). The questionnaires were mailed out between 20 ^th^ January 2020 and 5 ^th^ January 2022. Of participants sent a 3-month questionnaire, 144 (62.9%) were female (pen group n=66, 59.5%; no pen group n=78, 66.1%), the mean (SD) age was 73.2 (5.9) years (pen group 72.6 (5.5); no pen group 73.7 (6.2)), and 14 (6.1%) had been randomised to receive GBP 5 and/or a pen in the factorial recruitment SWAT (pen group n=7, 6.3%; no pen group n=7, 5.9%). 

The proportion of participants who returned their 3-month questionnaire was similar in the two groups (pen n=107, 96.4%; no pen n=117, 99.2%) ( Table 2). There was no evidence of a difference in return rates between the two groups (OR 0.23, 95% CI 0.02 to 2.19, p=0.20). The adjusted difference in proportions was -2.6 percentage points (95% CI -6.4 to 1.1).

**Table 2.** Summary of SWAT trial results (ITT analysis).

Results				
Pen	No Pen	Overall		
**Returned 3-month questionnaire, n/Total (%)**			**OR (95% CI)**	**p-value**
107 / 111 (96.4)	117 / 118 (99.2)	224 / 229 (97.8)	0.23 (0.02, 2.19)	0.20
**Reminder sent, n/Total (%)**			**OR (95% CI)**	**p-value**
30 / 111 (27.0)	35 / 118 (29.7)	65 / 229 (28.4)	0.85 (0.48, 1.53)	0.60
**Time to response (days), median (95% CI)**			**HR (95% CI)**	**p-value**
22.0 (13.0, 24.0)	21.0 (13.0, 24.0)	22.0 (14.0, 23.0)	0.91 (0.69, 1.18)	0.47
**Number of completed items (if questionnaire ** **returned), mean (SD)**			**Mean difference ** **(95% CI)**	**p-value**
77.2 (1.4)	76.6 (2.6)	76.9 (2.1)	0.51 (−0.04, 1.06)	0.07

**Figure 2.**
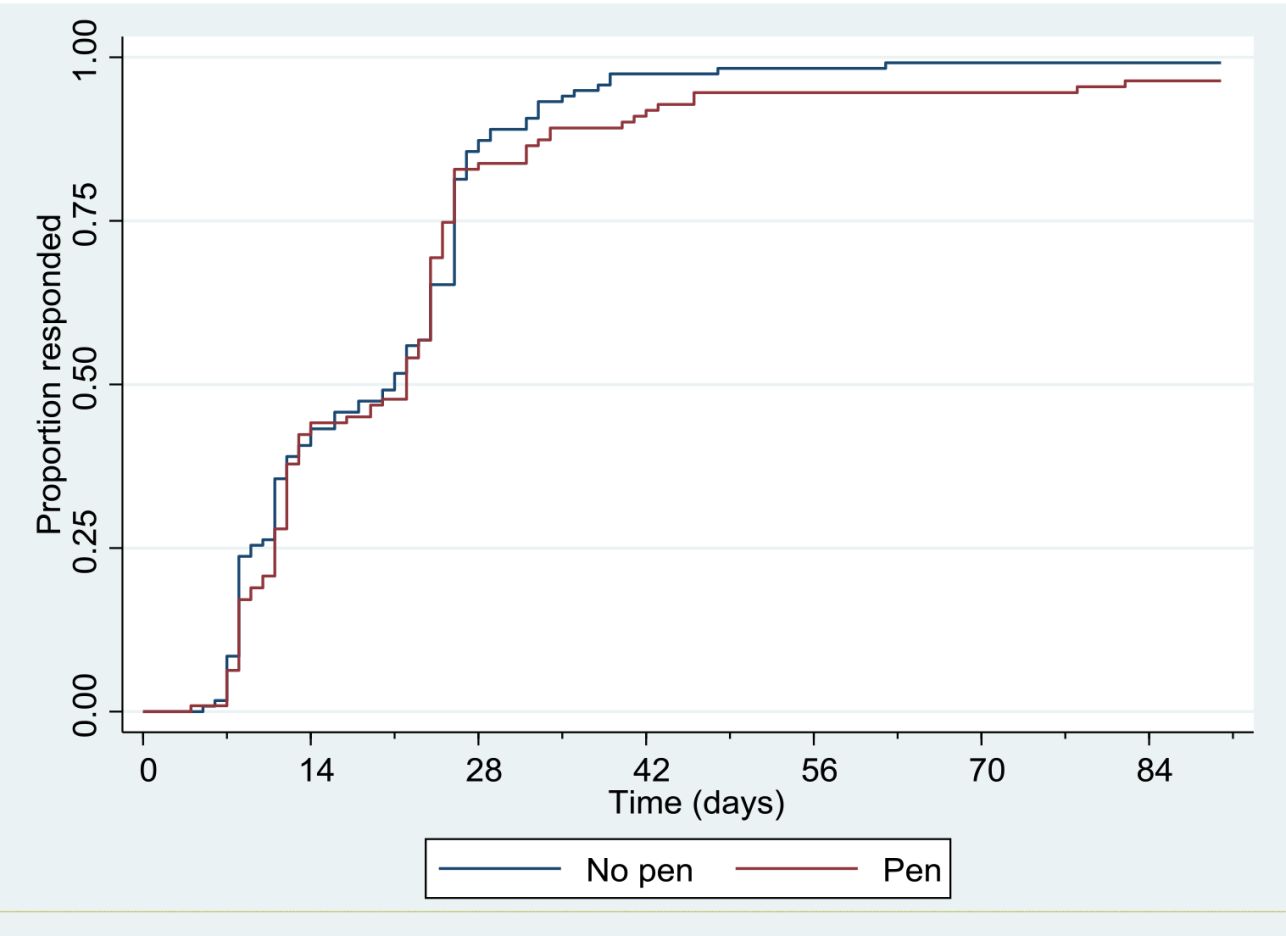
Kaplan–Meier survivor functions for time to return 3-month follow up questionnaire.

There was no evidence of a difference in the proportion of participants sent a reminder in each of the groups (pen n=30, 27.0%; no pen n=35, 29.7%; OR 0.85, 95% CI 0.48 to 1.53, p=0.60), nor in the time to return the questionnaire. The median time to return was 22 days in the pen group and 21 days in the no pen group (HR 0.91, 95% CI 0.69 to 1.18, p=0.47) ( Figure 2).

While the Grambsch and Therneau ^18^ test provided no evidence that the proportional hazards assumption had been violated (covariate-specific test for SWAT allocation p=0.56; global test p=0.56), the lines in the Kaplan–Meier curve for the time to return between the two groups cross one another, which can be an indication that the proportional hazards assumption is unsafe. The log-rank test and generalized gamma AFT model conducted as post hoc sensitivity analyses did not indicate evidence of a difference between the two groups (log-rank test: χ ^2^(1)=0.67, p=0.41; AFT model: time ratio 1.05, 95% CI 0.90 to 1.23, p=0.56).

Among participants who returned a questionnaire, there was weak evidence of a difference in the number of items on the questionnaire completed between the two groups (mean (SD): pen 77.2 (1.4); no pen 76.6 (2.6), mean difference 0.51, 95% CI −0.04 to 1.06, p=0.07). However, there was evidence of a ceiling effect with this outcome, with 64.6% (n=148) of returned forms having 100% completion (response to all 78 items). Additionally, there was a difference in the variance between the two groups with a larger SD in the no pen group than the pen group. This was caused by a small number of participants completing noticeably fewer items in the pen group than the no pen group (range 62–78 compared to 72–78). The standardised residuals from the linear regression demonstrated deviation from normality and so a post hoc Wilcoxon rank-sum test was performed, which indicated some evidence of a difference (p=0.09).

### Per-protocol analysis

A total of 202 participants were included in the per-protocol analyses (pen n=84; no pen n=118). Among these, 129 (63.9%) were female (pen group n=51, 60.7%; no pen group n=78, 66.1%), the mean (SD) age was 73.7 (6.0) years (pen group 73.7 (5.8); no pen group 73.7 (6.2)), and 11 (5.5%) had been randomised to receive GBP 5 and/or a pen in the factorial recruitment SWAT (pen group n=4, 4.8%; no pen group n=7, 5.9%). Results are provided in Table 3 and are similar to the ITT analysis. 

**Table 3.** Summary of SWAT trial results (PP analysis).

Results				
Pen	No Pen	Overall		
**Returned 3-month questionnaire, n/Total (%)**			**OR (95% CI)**	**p-value**
82 / 84 (97.6)	117 / 118 (99.2)	199 / 202 (98.5)	0.42 (0.04, 4.91)	0.49
**Reminder sent, n/Total (%)**			**OR (95% CI)**	**p-value**
22 / 84 (26.2)	35 / 118 (29.7)	57 / 202 (28.2)	0.85 (0.45, 1.59)	0.60
**Time to response (days), median (95% CI)**			**HR (95% CI)**	**p-value**
13.0 (12.0, 20.0)	21.0 (13.0, 24.0)	16.0 (13.0, 22.0)	0.99 (0.75, 1.33)	0.97
**Number of completed items (if questionnaire** **returned), mean (SD)**			**Mean difference** **(95% CI)**	**p-value**
77.0 (1.5)	76.6 (2.6)	76.8 (2.2)	0.37 (−0.26, 0.99)	0.25

### Meta-analysis

**Figure 3.**
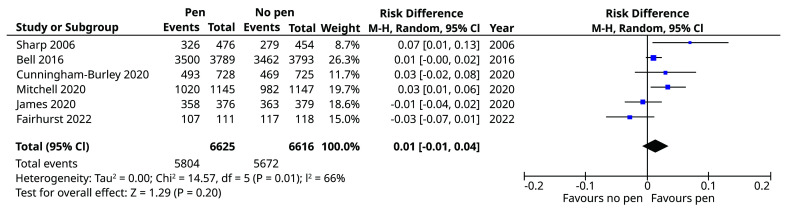
Meta-analysis of inclusion of a pen on questionnaire return rates.

Details of the included studies are as follows. Bell *et al.* (2016) ^6^ evaluated the use of adding a pen to the 60-month questionnaire in a trial of screening for the prevention of fractures in women aged 70–85 years; in Cunningham-Burley *et al.* (2020) ^7^, the pen was added to the 14-week questionnaire in a slip-prevention trial among NHS staff (mean (SD) age 43 (11.3) years); James *et al.* (2020) ^8^ enclosed the pen in the 12-month questionnaire in a falls prevention trial in older people (65 years+); Mitchell *et al.* (2020) ^9^ investigated pens for the 14-week questionnaire in an orthopaedic trial (mean (SD) age 69 (8.9) years); and Sharp *et al.* (2006) ^10^ embedded the pen SWAT in a cervical screening trial in women (mean (SD) age 34 (10.4) years) at their next follow-up (12, 18, 24, or 30 months). A random effects meta-analysis conducted using RevMan 5.3 (RRID: SCR_003581) indicated that the pooled effect across the six included studies was a risk difference, favouring use of a pen, of 1% (95% CI -1% to 4%, p=0.20; Figure 3). An I ^2^ value of 66% indicates moderate to large heterogeneity.

## Discussion

The results of this trial do not indicate any demonstrable benefit of including a trial-branded pen with the postal 3-month questionnaire in the GYY trial. Indeed, a slightly higher response rate was observed in the no pen arm, albeit this required a marginally higher proportion of participants to be sent a reminder notice than in the pen group. The scope for improvement in the return rate for the questionnaire was extremely limited given that, in the no pen group, all but one participant who was sent a questionnaire returned it. Furthermore, because of the high rate of return in the control group, the trial was severely underpowered to be able to detect a difference and so we would not have expected any statistically significant results.

In the meta-analysis, two trials were observed to have a negative effect, ours and James *et al.* (2021); in both of these, the overall response rate was over 95%, whereas response rates averaged 78% among the four positive component trials. This may explain some of the heterogeneity observed, and further evidence the limited potential for improvement when the response rates are already high. To address these limitations, future meta-analyses could include factors to correct for baseline response rates, allowing the impact of consistently high response rates to be accounted for.

Follow-up in GYY straddled the outbreak of the COVID-19 pandemic. A quarter of the 3-month questionnaires were sent out prior to COVID-19 having any real presence in our daily lives (all in January 2020), the next 3-month follow-ups were only due in December 2020 or later (up to January 2022). An exploratory, *post hoc* examination of the data suggests response rates were higher, across both the pen and no pen groups, in the follow-ups sent during the pandemic (97.7% and 100%, respectively) than those sent before (91.7% and 96.9%, respectively). This may be a chance finding, or it is possibly a direct consequence of the pandemic. Participants, particularly given their age, were likely to be adhering to social isolation guidelines and so may have had more time at home to complete the questionnaire. Additionally, it is feasible that news coverage of the pandemic could have increased awareness and respect in the population of the importance of research, trials and data, thus leading to greater engagement in the trial. The continually high response rates might additionally be attributed to the age group of participants, with many likely to be out of full-time employment or retired, hence able to more easily allocate time to completing and returning questionnaires, despite their reasonable length (the 3-month questionnaire was 16 pages long). 

The strength of this study was that it was a randomised trial; however, since it was conducted in a population of older adults with multimorbidity, and particularly during the COVID-19 pandemic, findings may not be generalizable to other populations or contexts. This trial already implemented several retention strategies including sending an SMS to participants a few days before their postal questionnaire arrived, including an unconditional GBP 5 ‘thank you’ payment, and reminder questionnaires and phone calls. All of these may have lessened the potential benefit of the addition of a pen with the mail out. Also, the incentive was tested at a reasonably early timepoint in the trial (3 months), when engagement in the trial might still be expected to be high; perhaps an increased benefit would have been seen at a later timepoint (further follow-ups in GYY were conducted at 6 and 12 months).

## Conclusion

This SWAT suggests that enclosing a pen in a questionnaire mail out may not be an effective method to increase response rates in a trial of older adults with multimorbidity, particularly when other initiatives are in place, such as a prenotification SMS, an unconditional financial incentive, and a robust reminder procedure as was the case in this trial. Nevertheless, this SWAT adds to the growing evidence base of the effect of sending a pen out to trial participants on the rate of retention. Current pooled evidence suggests pens may still offer an effective incentive for improving response rates.

## Data Availability

OSF: Underlying data for ‘Enclosing a pen in a postal questionnaire follow-up to increase response rate: a Study within a Trial’. https://doi.org/10.17605/OSF.IO/26MPA This project contains the following underlying data: Data file 1: GYY_retention_SWAT_csv_data.csv Data file 2: GYY_retention_SWAT_Stata_data.dta Data are available under the terms of the Creative Commons Zero “No rights reserved” data waiver (CC0 1.0 Public domain dedication). OSF: Code to replicate the completed analyses in ‘Enclosing a pen in a postal questionnaire follow-up to increase response rate: a Study within a Trial’. https://doi.org/10.17605/OSF.IO/26MPA Code file: GYY_retention_SWAT_analysis.do Analysis code, revised following peer-review, can be found here: https://doi.org/10.17605/OSF.IO/9BGW6 Code file: GYY_retention_SWAT_analysis_revised.do Data are available under the terms of the Creative Commons Zero “No rights reserved” data waiver (CC0 1.0 Public domain dedication). OSF: CONSORT checklist for ‘Enclosing a pen in a postal questionnaire follow-up to increase response rate: a Study within a Trial’. https://doi.org/10.17605/OSF.IO/26MPA Data are available under the terms of the Creative Commons Zero “No rights reserved” data waiver (CC0 1.0 Public domain dedication).
