# Supplementary material for: Enclosing a pen in a postal questionnaire follow-up to increase response rate: a study within a trial
Source: NIHR Open Res. 2023 Apr 20;2:53. Originally published 2022 Oct 31. [Version 2] doi: 10.3310/nihropenres.13324.2 (PMC7614271; doi:10.3310/nihropenres.13324.2)
Supplement: Peer review history

# Reviewer report: Reviewer response for version 2

**DOI:** 10.3310/nihropenres.14559.r29183

**Published:** 2023-04-20

**Authors:** Graeme MacLennan

**Affiliations:** 1 Institute of Applied Health Sciences, University of Aberdeen, Aberdeen, Scotland, UK

No further comments from me.

Is the work clearly and accurately presented and does it cite the current literature?

Yes

If applicable, is the statistical analysis and its interpretation appropriate?

Yes

Are all the source data underlying the results available to ensure full reproducibility?

Yes

Is the study design appropriate and is the work technically sound?

Yes

Are the conclusions drawn adequately supported by the results?

Yes

Are sufficient details of methods and analysis provided to allow replication by others?

Yes

Reviewer Expertise:

Clinical trials statistician

I confirm that I have read this submission and believe that I have an appropriate level of expertise to confirm that it is of an acceptable scientific standard.

# Reviewer report: Reviewer response for version 1

**DOI:** 10.3310/nihropenres.14450.r29002

**Published:** 2023-02-20

**Authors:** Valarie Smith

**Affiliations:** 1 School of Nursing and Midwifery, Trinity College Dublin, Dublin, Ireland

This is a well-written paper that adds to the body of evidence on the use of a pen for the return of follow-up questionnaires in a trial. The study is presented as a SWAT and is methodologically sound. I enjoyed reading the paper, and I liked that the authors updated the previous meta-analysis on this topic to incorporate their own results. I only have two queries regarding the report:

1. The authors note the statistician was not blinded. Why was this? An explanation would help.
2. Also in the description of the intervention, I wasn't clear where the 5GBP came in. Did all participants of the main host trial (both groups) receive this, or was this specific to this (or another) embedded SWAT? Again a line or two for reader clarity would be helpful here.

Is the work clearly and accurately presented and does it cite the current literature?

Yes

If applicable, is the statistical analysis and its interpretation appropriate?

Yes

Are all the source data underlying the results available to ensure full reproducibility?

Yes

Is the study design appropriate and is the work technically sound?

Yes

Are the conclusions drawn adequately supported by the results?

Yes

Are sufficient details of methods and analysis provided to allow replication by others?

Yes

Reviewer Expertise:

RCTs, SWATs, trial methodology

I confirm that I have read this submission and believe that I have an appropriate level of expertise to confirm that it is of an acceptable scientific standard.

# Response

**Published:** 2023-04-04

**Authors:** Caroline Fairhurst

**Affiliations:** University of York, UK, UK

We would like to thank the reviewer for their thorough and constructive feedback. We outline below our responses and the changes made to the manuscript, which we believe have really improved the paper.

 We have added further details to the Blinding section to explain the justification for the absence of blinding of the trial statistician in this SWAT, as this was considered low risk as the outcomes were subjective and were prespecified in the host trial protocol.

 The Intervention section has been amended to clarify the use of the GBP 5 incentive. This was not specific to a SWAT but formed part of the host trial’s approach to encouraging continuing participation. As such, the inclusion of GBP 5 was used at each follow-up timepoint across both the intervention and control arms.

# Reviewer report: Reviewer response for version 1

**DOI:** 10.3310/nihropenres.14450.r28906

**Published:** 2023-01-23

**Authors:** Graeme MacLennan

**Affiliations:** 1 Institute of Applied Health Sciences, University of Aberdeen, Aberdeen, Scotland, UK

This manuscript describes the results of a randomised SWAT to evaluate the inclusion of a pen to with a questionnaire to improve response the Gentle Years Yoga trial. My review is primarily statistical, I have only minor comments and suggestions:

- Sample size, suggest that authors make clear that the 80% control response is the based on the 20% expected attrition in GYY (if indeed it was, could be a coincidence!).
- Methods/Results, the primary outcome is reported on the adjusted absolute difference scale also, this is not mentioned in the methods, I take it this was estimated from the logistic regression model? I ask because the upper bound of that CI is not possible given the control proportion.
- Methods/results, the description of the post hoc analysis would be better in the methods section. As an aside, rather than the PH assumption being violated, I think the the more reasonable assumption is that underlying DGM is a HR of 1, but accept the belt and braces approach. 
- Number of items completed, there are ceiling effects here and clear difference in variance, probably caused by radically fewer items completed by one or two people in the control group. Did you investigate this further? Would the primary outcome completion at 3 months not have been of more interest?
- Meta-analysis, as aside, I agree with the point in discussion about high response rates in the controls groups, which points towards more sophisticated MA requirements in future (correcting for baseline risk).

Suggestions

- In this instance the time-to-event outcome might be better plotted as the "failure", i.e. proportion responding, rather than yet to respond, this makes more intuitive sense. KM plot, the ticks on the time axis could be weekly or fortnightly, again more intuitive for this time scale. This is just a suggestion, can be ignored. 

Is the work clearly and accurately presented and does it cite the current literature?

Yes

If applicable, is the statistical analysis and its interpretation appropriate?

Yes

Are all the source data underlying the results available to ensure full reproducibility?

Yes

Is the study design appropriate and is the work technically sound?

Yes

Are the conclusions drawn adequately supported by the results?

Yes

Are sufficient details of methods and analysis provided to allow replication by others?

Yes

Reviewer Expertise:

Clinical trials statistician

I confirm that I have read this submission and believe that I have an appropriate level of expertise to confirm that it is of an acceptable scientific standard.

# Response

**Published:** 2023-04-04

**Authors:** Caroline Fairhurst

**Affiliations:** University of York, UK, UK

We would like to thank the reviewer for their thorough and constructive feedback. We outline below our responses and the changes made to the manuscript, which we believe have really improved the paper.

 The SWAT was not specifically powered to detect a certain difference and so the Sample Size section simply presents an example of the difference that the trial would be powered to detect. The 80% control rate is not necessarily based on the expected attrition assumed in the sample size calculation for the host trial as this was based on participants who provide a valid response to the EQ-5D-5L at one or more of the assessment timepoints in the host trial (3, 6, and 12 months), not necessarily at a single timepoint.

 We have added into the Methods section that we present the treatment effect for the primary outcome in the form of an adjusted absolute difference in proportions, as well as an odds ratio, and clarified that these are both estimated from the primary logistic regression model. We have also moved the description of the post hoc sensitivity analyses from the Results section to the Methods. We have changed the Kaplan-Meier graph as suggested; we agree that the proportion responding is a more appropriate measure to display given the context of the outcome and a scale with increments every 7 days (labels every 14) is more intuitive and readable for the user.

 We did not specify the completion of the primary outcome (from the host trial, the EQ-5D-5L) as a binary outcome for this SWAT. This was because the EQ-5D-5L is a short, standardised instrument and was the first section in the questionnaire and, as such, we expected that if a questionnaire was returned then this section would be well completed. Therefore, this analysis would have been virtually identical to the primary analysis of whether or not a questionnaire was returned. We opted to consider the number of completed items in total as, in addition to several standardised instruments, this also included questions asking about recent falls, health resource use, and participation in yoga over the previous 3 months, which may not have been completed as thoroughly. The reviewer is correct that there is evidence of a ceiling effect with this outcome, and that the difference in variance (the standard deviation was smaller in the ‘pen’ group than the ‘no pen’ group) was due to fewer items completed by a small number of the respondents in the ‘no pen’ group than the ‘pen’ group. On reflection, we agree that this warranted further investigation. Therefore, we have added in more details about the distribution of this outcome into the Results, and now include a post hoc Wilcoxon rank-sum test, which is appropriate when the assumption of linear regression that the residuals are normally distributed is questionable.

 We have also expanded on recommendations for future meta-analyses to correct for baseline risk in the Discussion section.
